# A web-based psychoeducational simulation game for adults in stepfamilies (*GSteps*)—study protocol for a randomized controlled feasibility trial

**DOI:** 10.3389/fpsyg.2022.1020979

**Published:** 2022-11-24

**Authors:** Carina Mota Santos, Maria Emília Costa, Brian Jensen Higginbotham, Mariana Veloso Martins

**Affiliations:** ^1^Faculty of Psychology and Education Science, University of Porto, Porto, Portugal; ^2^Center for Psychology, Faculty of Psychology and Education Science, University of Porto, Porto, Portugal; ^3^Utah State University, Logan, UT, United States; ^4^Emma Eccles Jones College of Education and Human Services, Utah State University, Logan, KS, United States

**Keywords:** remarriage, stepfamilies, web-based intervention, psychoeducational game, behavior modeling, randomized controlled trial, study protocol

## Abstract

**Background:**

Stepfamilies are a prevalent family form. However, less stable than nuclear, first marriage families due to the presence of risk factors such as the absence of social norms and the presence of stepchildren. Stepfamilies have unique educational needs regarding stepparenting and co-parenting issues. The development and documentation of psychoeducational intervention strategies can facilitate dissemination of ongoing studies and promote transparency. This article describes the background, design and protocol of a randomized controlled trial (RCT) evaluating the eficacy and feasibility of a web-based Psychoeducational Simulation Game (*GSteps*). Behavior-modeling video training (BMT) is used to demonstrate and promote relational skills, stepparenting and co-parenting effective strategies for adults in stepfamilies. A mental health professional will be available within the *GSteps* platform for clarification or emotional support.

**Methods/design:**

A RCT design is presented to evaluate the outcomes of a self-administered, interactive and web-based psychoeducational Game targeting dyadic marital adjustment and interpersonal skills as the primary outcomes and remarriage beliefs, family function and stepparenting and co-parenting attitudes as the secondary outcomes. Other outcome measures include satisfaction with *GSteps*, participants’ knowledge learned after the intervention and a purposive sampling method will be used to access feasibility. The minimum required sample size is 112 participants (56 per condition) randomly allocated either to an experimental group (EG), receiving *GSteps* intervention, or to a wait-list control group (CG). A survey is conducted electronically. Assessments take place at baseline (*T_0_*), after the intervention (*T_1_*) and 1-month follow-up (*T_2_*).

**Discussion:**

This protocol presents a RCT aimed at evaluating the efficacy of a web-based psychoeducational intervention (*GSteps*) designed for improving marital, stepparenting and co-parenting skills in adults who live in stepfamilies. The use of the protocol and results of intervention studies may guide the use and refinement of web-based psychoeducational intervention for stepfamilies. Additionally, *GSteps* may become a tool for health professionals to enhance stepfamily functioning, stepparenting skills, and marital adjustment of remarried adults.

## Introduction

### Background

Remarriage has been characterized as a *phenomenon* since the 1960s (Schlesinger, 1968). Although the rate of remarriages has been declining steadily in recent decades for both men and women (Schweizer, 2019), cohabitation has become an increasingly common lifestyle choice. A growing proportion of cohabiting unions are second unions that form stepfamilies when there are stepchildren (Manning, 2015). Between 2007 and 2016, the rate of unmarried stepcouples in cohabitation increased by 29% (Stepler, 2017). This trend has been observed both in America and in European countries (Higgins et al., 2020), reflecting the increasing social acceptability of unmarried couples and families (Guzzo, 2018).

Although the literature identifies differences between remarried unions and cohabiting unions (e.g., Brown, 2006), both are less stable than nuclear, first marriage families (Cherlin, 2017). This instability has been explained by the absence of social norms about the functioning of families with stepchildren (Nock, 1995; Kelly and Ganong, 2011; Coleman et al., 2022). Nuclear families have socially prescribed norms and expectations (e.g., the parent role is clear within any biological family) that “institutionalizes” their family form. The absence of social norms, the ambiguity of roles and functions of stepfamily members (remarried and cohabiting) means that these families are not completely institutionalized (Cherlin, 1978) and contribute to a negative impact on stepfamily dynamics and marital relationship (Garneau and Pasley, 2017).

Other factors can also contribute to the instability of stepfamilies. For example, stepcouples have to simultaneously deal with the tasks related to couple formation and parenting, often with children in different developmental stages (Dupuis, 2007). Although much has been investigated about the intertwinement between stepcouples’ functioning and stepfamily functioning (e.g., Papernow, 2013), intervention programs designed for stepcouples are relatively scarce (Adler-Baeder and Higginbotham, 2004). Those available are mostly focused on stepparenting issues and use traditional education formats, with a facilitator moderating multiple group sessions (Nicholson et al., 2007).

Effective interventions to enhance stepcouples’ dynamics should address not only factors that are unique to stepparenting, but also factors that are inherent to the couple dyad (Halford et al., 2003; Adler-Baeder and Higginbotham, 2004). In common with other couple types, couples in stepfamilies may consider programs that promote general relationship skills such as communication training (e.g., Ahrari et al., 2020), problem-solving (e.g., Babcock et al., 2013), empathy skills (e.g., Adler-Baeder, 2007), conflict and stress management and building friendship and affection (Gottman, 1999). But for educational programs targeting stepcouples, it is important to consider their unique challenges. First, remarried people often face unrealistic thoughts around the notion that “the new partner should be perfect and better than previous one” (Higginbotham and Adler-Baeder, 2008a). Second, it is frequent to carry unresolved emotional patterns from previous marriage(s) to remarriage, such as feelings of guilty, betrayal or loss (Fredericson and Handlon, 1994; Faber, 2004). Third, after a previous marital dissolution, remarried partners feel social pressure to succeed (Fredericson and Handlon, 1994; Bernstein, 2000). Fourth, besides social pressure, the social network has to be rebuilt and there is a tendency to perceive lower levels of social support (Bradbury et al., 2000; Ganong and Coleman, 2017; Dainton, 2019). Fifth, the management of financial resources can be connected to, or dependent on, the economic decisions of former partners (e.g., complying with child support; Ganong and Coleman, 2017).

The interaction between parenting, co-parenting and stepparenting (with potential spillover effects in the quality of the relationship) should be considered when designing a program for stepcouples. Previous research has shown that the presence of unrealistic myths or expectations (e.g., “instant love” between stepparent and stepchild; Higginbotham and Agee, 2013; Santos et al., 2022), may strain stepfamilies. Overall, these myths are based on nuclear family ideology, where love is usually an automatic and unquestioned feeling (Ganong and Coleman, 2017). On the other hand, the myth that stepparents and stepchildren can never learn to love each other can lead to other difficulties in building a positive relationship (Coleman et al., 1994; Ganong and Coleman, 2017).

Difficulties in roles definition and early imposition of discipline actions in the stepchild’s rearing can also contribute to unhealthy relationships (Papernow, 1993; Adler-Baeder, 2007). Thus, to develop a healthy stepparenting, it is recommended that couples (1) developing realistic expectations (Fine and Kurdek, 1994; Higginbotham and Adler-Baeder, 2008b); (2) empathize by validating stepchildren’s feelings and emotions (Adler-Baeder, 2007; Agulhas and Anciães, 2018); (3) discuss with partner about stepparent role (Adler-Baeder, 2007; Papernow, 2013); (4) engage in cooperative parenting instead of trying to “replace” the non-residential parent (Adler-Baeder, 2007; Dupuis, 2007; Papernow, 2013); (5) recognize that the ex-spouse will always be part of stepfamily (Papernow, 2013); (6) utilize healthy co-parenting practices between ex-spouses protecting children from the details of divorce process (Pringle and Ehrenberg, 2005; Adler-Baeder, 2007; Pringle, 2008), parental conflict and loyalty conflicts (Papernow, 1993; Adler-Baeder, 2007; Agulhas and Anciães, 2018). These practices minimize children’s rejection behaviors; enhance positive stepfamily functioning; promote the construction of a unique stepfamily identity through the emotional connection of stepfamily members and increase marital quality and satisfaction (Papernow, 1993; Adler-Baeder and Higginbotham, 2004; Adler-Baeder, 2007; Gelatt et al., 2010).

### Developing a theory-based online educational game for promoting relational skills in stepcouples

Some non-traditional efforts have piloted ways to help stepcouples prevent marital and family difficulties (e.g., online intervention; telehealth; Braithwaite and Fincham, 2009; Gelatt et al., 2010). Web-based self-administered interventions with a behavior-modeling training (BMT) approach (i.e., visual demonstrations of behaviors) appear to increase self-efficacy and motivation (Cairncross and Mannion, 2001). BMT promote preparation for practice by visualizing the performance of certain behavior (Taylor et al., 2005). This approach is based on Bandura and Walters’ social-learning theory and has been shown to be effective in producing sustainable skill improvement and behavior change with video modelling in parenting intervention programs (Glang et al., 2007). According to that theory, human thought and behavior are influenced not only by real experience but also by direct observation. Bandura and Walters (1977) also concluded that learning is most effective when people observe the consequences of engaging in a specific behavior. Combined with interactive teaching aids, BMT allows individuals to access to learning environments to ‘practice’ problem-solving skills and critical thinking in a virtual simulation that replicates real-life problematic situations (Homanova et al., 2019). Additionally, there is evidence that self-administrated web-based programs can be more effective than face-to-face group sessions (U.S. Department of Education, 2009). Taylor et al. (2005) conducted a meta-analysis of 117 studies of adult training programs and concluded that BMT was effective in producing sustainable skill improvement and post-training behavior change. Attempts to include technology’ advances to differentiate the training modalities have been increasing, especially due to the pandemic situation, but randomized controlled trial (RCT) studies that investigate stepfamily outcomes remain limited (Gelatt et al., 2010).

Prevention and psychoeducational programs for stepfamilies generally provided positive effects (Whitton et al., 2008). A meta-analysis of 14 studies conducted by Lucier-Greer and Adler-Baeder (2012) concluded that education programs for stepcouples had large effects in parenting and family functioning. *Smart Steps Program* (Higginbotham and Adler-Baeder, 2008b) was one of the education programs evaluated by these authors. They concluded that *Smart Steps* increased relationship skills, stability, and commitment for stepparents and these improvements endured 1 month after the study. Clinical programs for stepfamilies have also shown similar positive results. Behavioral family intervention (Nicholson and Sanders, 1999) or emotionally focused family therapy (Furrow and Palmer, 2007) were two different interventional approaches that demonstrated greater reductions in couple conflict over parenting practices and promote stability, cohesion and attachment in the developing stepfamily system.

The web-based, interactive training programs for couples in stepfamilies (e.g., Parent et al., 2019), in particular those using BMT (Trone, 2002; Gelatt et al., 2010) have also demonstrated promising results. Specifically, Trone (2002) reported higher levels of family adjustment in families with a stepfather after the intervention. Gelatt et al. (2010) documented significant effects in stepparenting, stepfamily, and couple domains, with both parents and stepparents increasing their skills. However, these benefits should be interpreted with caution as several limitations were noted, including the lack of random assignment, control-group, and pre-, post-, and follow-up assessments (e.g., Parent et al., 2019). These available web-based programs are self-administered (Gelatt et al., 2010) and do not provide background support from health professionals (e.g., psychologist; Gelatt et al., 2010). Besides that, these programs also do not address the many unrealistic expectations so common in stepfamilies that contribute to high levels of marital and parental dysfunction (Higginbotham and Adler-Baeder, 2008a; Higginbotham and Agee, 2013). Furthermore, to the authors’ knowledge, no intervention tools (traditional or web-based) have yet been developed for Portuguese stepfamilies.

### Aims

To fill the gaps in the literature, our protocol intends to evaluate the efficacy and feasibility of a new web-based psychoeducational intervention (*GSteps*). The protocol outlines a comparison with a non-intervention control condition in a sample of Portuguese speaking adults in stepfamilies (parents and stepparents). The protocol offers options to examine changes in *stepparenting and co-parenting attitudes, remarriage beliefs, dyadic marital adjustment, marital social skills* and *family function.*

## Methods and analysis

### Study design

**Figure 1:**
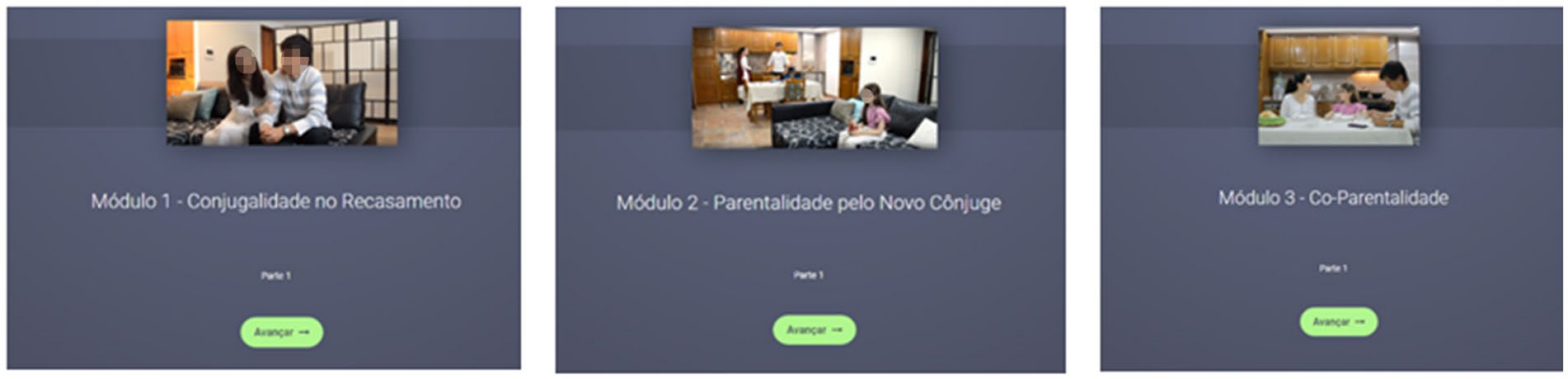
The three Game modules of *Gsteps* − Module 1: onjugality; Module 2: Stepparentimg; Module 3: Co-parenting.

**Figure 2:**
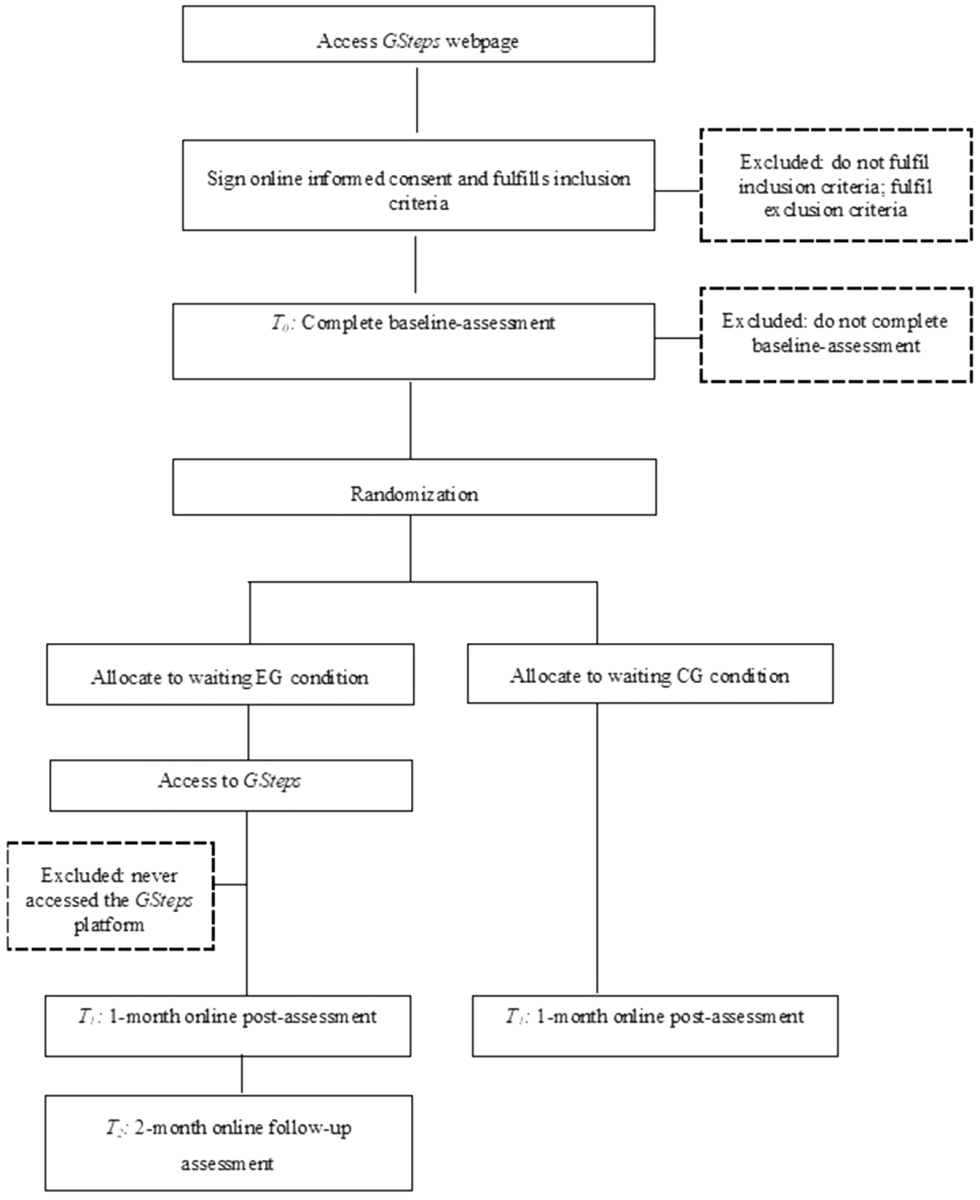
Study schedule of enrollment, intervention and assessments.

This study protocol is a two-arm double-blind prospective RCT comparing a web-based psychoeducational intervention to a waiting control condition among adults in stepfamilies (*N* = 112). The proposed intervention consists of a three-module (Figure 1) interactive Game that lasts a minimum of 30 and a maximum 60 min and can be played over the course of 1 month in computer. Modules are sequential and focus on the romantic relationship, co-parenting and step-parenting. Assessments are made before (*T_0_*) and 1 month after the intervention (*T_1_*). The experimental group (EG) has a second follow-up after 2 months (*T_2_*). The control group (CG) is on a waiting list until completion of *T_1_*, and then gets access to the intervention. Intervention and measurements are carried out online. The protocol uses the learning app H5P and *Limesurvey* survey design tool integrated into Moodle e-learning management system. The use of H5P interactive teaching aids to solve problems has been studied in literature as an important tool for educational context (Wang et al., 2016; Sinnayah et al., 2021). Figure 2 displays the study schedule of enrollment, interventions and assessments. This RCT will follow the SPIRIT guidelines (Chan et al., 2013a, 2013b) and the CONSORT statement (Moher et al., 2010; Schulz et al., 2010).

### Instruments and measures

A sociodemographic questionnaire will be used at *T_0_* to describe the sample and compare groups. Primary and secondary outcome measures administered at *T_0_* and *T_1_* will assess intervention efficacy and changes in marital quality, remarriage beliefs, co-parenting and stepparenting attitudes, and family functioning. Stability of these changes is assessed at *T_2_* for those in the EG. Specific outcomes related to the quality of the intervention and perceptions of intervention benefits will be accessed after the intervention for participants in the experimental condition group.

#### Sociodemographic questionnaire

Socio-demographic data is obtained through a questionnaire that included gender, date of birth, educational attainment, professional status, number of (step) children and financial and economic situation. The questionnaire will also include questions regarding respondent’s relationship history (pre-remarital status, type of divorce (when applicable), time spent between the previous and current relationships and length of the remarried relationship).

#### Primary outcome measures

##### Revised dyadic adjustment scale

The DAS-R (Busby et al., 1995; Portuguese validation by Pereira et al., 2017) is a self-rating questionnaire with 14 items designed to assess the dyadic marital adjustment through three dimensions: Consensus (items 1–6 rated on a Likert-type scale range between 5 - always agree to 0 – always disagree), Satisfaction (items 7–10 rated on a Likert-type scale range between 0 - all the time to 5 – never) and Cohesion (items 11–14 rated on a Likert-type scale range between 0 − never to 5 – more often). Higher scores indicate greater marital adjustment. The internal consistency of the overall scale in original version was 0.90 (Busby et al., 1995) and in a Portuguese validation it was 0.89 (Pereira et al., 2017).

##### Marital social-skills inventory

The MSSI (Villa and Del Prette, 2012; Portuguese version Aguiar et al., 2018) is a self-report measure that evaluates the frequency with which people present social behaviors that are of critical importance to a satisfactory marital relationship. Questionnaire has 17 items and four dimensions: Expressivity (item 10, 16 and 17; *α* = 0.83), Self-affirmation (item 1, 6, 7, 8, 9, 11, 12 and 14; *α* = 0.66), Self-control (item 4, 5, 13 and 15; *α* = 0.69) and Assertive Conversation (item 2 and 3; *α* = 0.55). Each dimension is rated on a Likert-type scale, ranging from definitely believe this is not true (1) to definitely believe this is true (5). Higher scores indicate greater marital social skills.

#### Secondary outcome measures

##### Stepparenting attitudes and beliefs

SAB can be obtained through a questionnaire developed for this protocol (see Appendix A) based on previous stepparenting cognition research (see Fine and Kurdek, 1994). Seventeen items are used to rate the participants’ *attitudes and beliefs* addressed in the program content, such as *“initially, discipline and authority in the child’s education should be imposed by the biological parent.”* on a 5-point scale ranging from 1 = *strongly disagree* to 5 = *strongly agree*. Higher scores indicate greater stepparenting positive attitudes and beliefs.

##### Co-parenting attitudes and beliefs

A specific questionnaire was developed for this protocol with nine items related to CAB based on the co-parenting belief inventory (Pringle and Ehrenberg, 2005; Pringle, 2008; see Appendix B). Items like *“Parents should not involve their children with details of the divorce process”* are rated on a 5-point scale ranging from 1 (*strongly disagree*) to 5 (*strongly agree*). Higher scores indicated greater co-parenting positive attitudes and beliefs.

##### Remarriage belief inventory

The RMBI (Higginbotham and Adler-Baeder, 2008a) can be used to assess participants’ beliefs regarding remarriage and stepfamilies in general. There are 19 items in the Portuguese version (Santos et al., 2022), distributed among seven subscales: (1) adjustment (4 items), (2) stepfamilies (2 items), (3) priority (3 items), (4) past (2 items), (5) partner (4 items), (6) success (4 items) and (7) finances (3 items). Each dimension is rated on a Likert-type scale, ranging from definitely believe this is not true (1) to definitely believe this is true (5). The Cronbach’s alpha for the total scale was in original version was 0.73 (for females) and 0.72 (for males; Higginbotham and Agee, 2013) and in Portuguese validation was 0.72 for the total scale (Santos et al., 2022). Higher scores indicated stronger remarital beliefs.

##### Systemic clinical outcome routine evaluation (SCORE-15)

The SCORE-15 (Stratton et al., 2010; Pereira, 2011; Vilaça et al., 2017) is a self-report questionnaire to provide an evaluation of family functioning with 15 items and three dimensions: Family strengths (FS), Family communication (FC) and Family difficulties (FD). Each dimension is rated on a Likert-type scale, ranging from “describe us: very well” (1) to “describe us: very bad” (5). The internal consistency of the overall scale in original version was 0.89 (Stratton et al., 2010) and in European Portuguese validation was 0.88 (Vilaça et al., 2017). Higher scores correspond to greater difficulties in family functioning.

#### Feasibility measures

An intervention fidelity plan will be conducted by accessing participants’ *GSteps* receptivity (e.g., satisfaction, usability, knowledge learned). Questions were based on previously feasibility measures used in context of web-based intervention that promoted positive parenting (Suárez et al., 2018). First, the *Program Satisfaction Scale (PSS)* will be used (Suárez et al., 2018) to provide an evaluation of satisfaction with intervention. This measure is a self-report questionnaire with 14 items and three dimensions: Usability (items 1, 2, 3, 4), Content (items 5, 6, 7, 8, 9), and Parenting impact (items 10, 11, 12, 13, 14). Usability dimension is rated on a Likert-type scale, ranging from “very difficult” (1) to “very easy” (5). Content and Parenting impact dimensions are rated on a Likert-type scale, ranging from “strongly disagree” (1) to “strongly agree” (5). Item 8 and 9 are edited for semantic adjustment due to the “Game” nature of the program.

Second, the *Intervention Perceived Benefit (IPB)* will be used to assess the participants knowledge learned after the intervention. Adapted from Adler-Baeder (2007), twelve questions were elaborated for this study (see Appendix C in online supplementary material) according to *GSteps* content. Responses must be given on a 5-point Likert scale, ranging from “strongly disagree” (1) to “strongly agree” (5). Higher scores correspond to greater knowledge learned.

Third, a purposive sampling method will also be used. This method is an intentional selection of participants based on their characteristics (Etikan et al., 2016) who are knowledgeable about a specific issue (Creswell and Plano Clark, 2011). Six participants from *EG* that completed the entire *GSteps* program (including follow-up assessment) will be selected to answer one open question related to participant’s experience - *Please describe your opinion whether the GSteps content realistically represents familiar situations that you have experienced*. These six participants will be (a) two individuals from complex stepfamilies (a man who is simultaneously father and stepfather and a woman who is simultaneously mother and stepmother) and (b) four individuals from simple stepfamilies. Two of them from a stepfather-family (a stepfather and a mother), and the other two from a stepmother-family (a father and a stepmother). In this way, we considered individuals from all possible configurations of stepfamilies, aiming to acquire more realistic feedback regarding their experience as *GSteps* “players.” Based on qualitative approach, IRAMUTEQ software (Lahlou, 2012; Camargo and Justo, 2013) will be used to conduct textual analysis.

## Procedure

### Recruitment and randomization

*GSteps* can be called an intervention, psychoeducational activity, or “Game.” Recruitment will be announced in social media (i.e., Facebook, Instagram, Self-help forums) or through a project website. On all these platforms, a link to an online questionnaire (*T_0_* – pre-test assessment) should be available. Participants meeting inclusion criteria and consenting to enter the study proceed and complete the pre-test questionnaire. After that, an automatic equation set will randomly (1:1) allocate participants to the EG or to the CG. Based on the random assignment, an automatic message is sent. Participants in the EG are invited to play *GSteps* and receive an email with a link and a confidential username and password. Participants allocated in CG receive an email informing them that they are on the waiting list. Both participants and the research team will be blind to the assignment.

### Eligibility criteria

All interested adults are required to complete screening questionnaires prior to randomization process. Inclusion criteria are (1) being in a stepfamily as a result of remarriage or repartnering; (2) length of current marriage/cohabitation of at least 6 months prior to enrollment; (3) having children from past relationships and/or stepchildren; (4) having access to a computer with internet connection; (5) to be native of the European Portuguese language. Subjects in a remarriage after widowhood (at least one of the partners is not divorced) are excluded. Furthermore, participants have to agree to participate *via* electronic informed consent and be willing to provide an e-mail address for contact during the study.

### *GSteps* development

**Figure 3:**
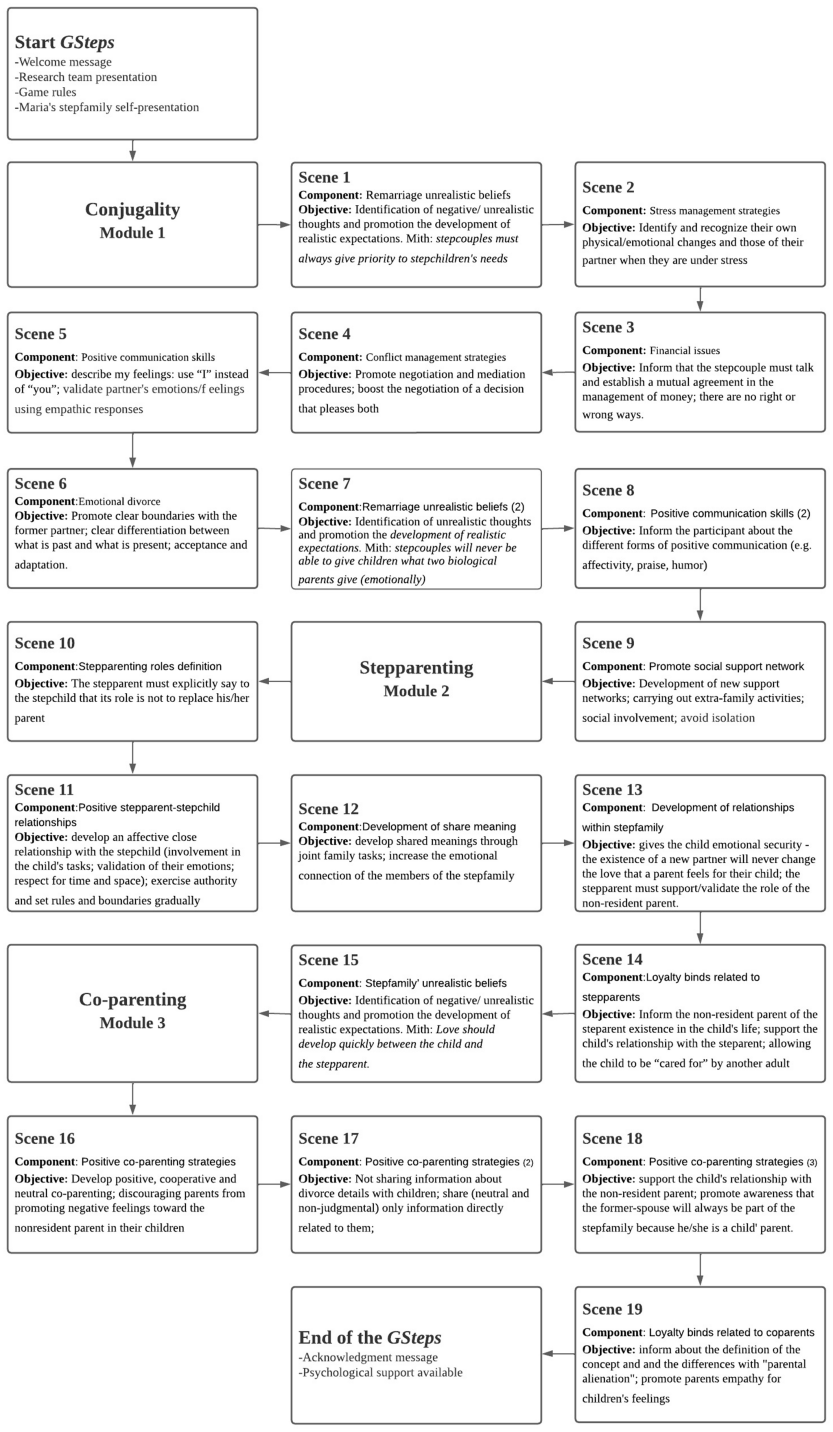
Branching scenario of the entire structure of *Gsteps.*

*GSteps* is a self-directed web-based interactive Game designed to increase stepparenting, co-parenting and marital skills for adults in stepfamilies. The initial phase of *GSteps* development involved an extensive literature review regarding healthy stepfamily functioning and satisfying stepcouple relationships (Santos et al., 2018; Santos et al., 2020). Factors unique to stepfamilies (e.g., stepparent-stepchild relationships) as well as factors that are common to all couples (e.g., communication skills) were used to build the *GSteps* intervention components. A total of 15 components were considered and incorporated into three content areas/modules: *Conjugality* – *Module 1* (emotional divorce, financial issues, positive communication skills, enhance the social support network, remarriage unrealistic beliefs, stress management strategies and conflict management strategies). *Stepparenting – Module 2* (positive stepparent-stepchild relationships, development of relationships within stepfamily, stepparenting roles definition, stepfamily’ unrealistic beliefs, loyalty binds related to stepparents and develop share meaning); *Co-parenting – Module 3* (positive co-parenting strategies and loyalty binds related to co-parents). The entire structure and contents of *GSteps* were demonstrated in Figure 3.

After determining the content of each topic and module, the developers dramatized fictional but real-life based narratives in a theater script to tell the story of a stepfamily that includes a mother (Teresa), an eight-year-old daughter (Maria), and a stepfather (António). These three characters were voluntarily represented by three actors from *Teatro Amador de Sandim* and gave us your written consent to record and release your image. Prior to dramatization, a spoken reflection was made separately including four volunteer target users: two interviewees (a stepmother and a stepfather) lived in a simple stepfamily (only one adult bring children from past relationships) and other two (a mother and a stepfather) lived in a complex stepfamily (both adults bring children from past relationships). We will collect feedback from subjects in different conditions (simple and complex) because the roles that they assume within the stepfamily are also different. In other words, in complex stepfamilies, both members of couple are simultaneously a parent (from their own children) and a stepparent (from partner’ children); but in simple stepfamilies the men and the women never have these two roles simultaneously.

Based on pilot feedback from the two major types of stepfamilies, changes in characters’ speeches, facial and body expressions were made to clarify the meaning of the content and dramatization. Before watching the videos, participants access the Game’s objectives and learn about the technical features. After that, the three actors present themselves as members of the stepfamily and their previous families’ history (e.g., stepfather is divorced and does not have kids). This information helps the participant to know the context of the presented family’s life cycle.

**Figure 4:**
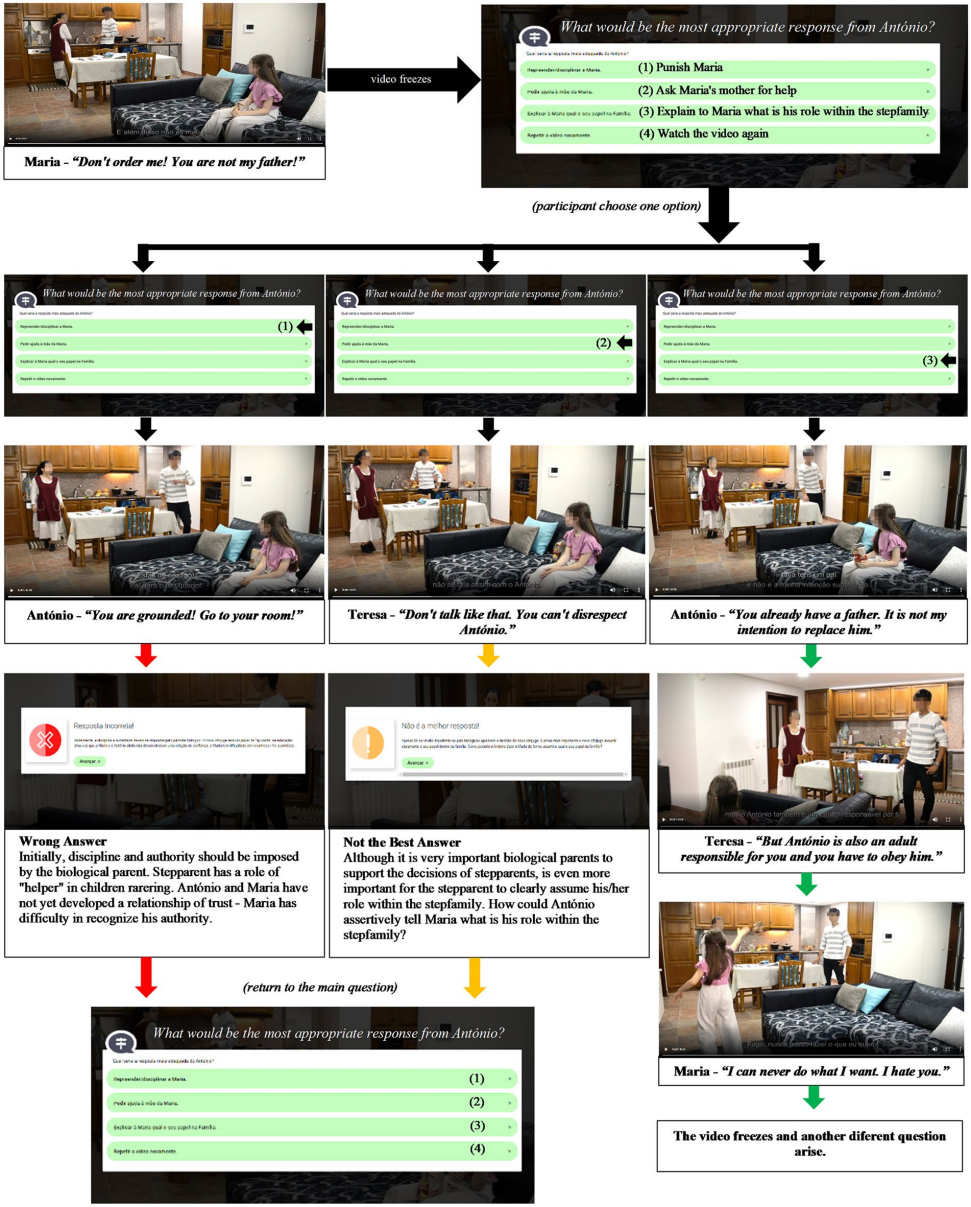
Branching scenario from Scene 10 of Module 2-“Stepparenting.”

Each module lasts between 10 and 20 min. Each is presented in a sequential video dramatization with some or all family members portraying relationship challenges (components). Videos freeze to ask participants which would be the better option in the face of a given conflict. Participants are able to see how their chosen option could play out. There are *right*, *wrong*, and *not the best* options, and a message pops up with a psychoeducational content focusing on positive and successful practices of stepparenting, co-parenting and remarital functioning to guide the participant to the right answer. All videos have subtitles to facilitate participants’ comprehension. For example, in module 2, the participant will see an argument between Maria and her stepfather on what Maria said – *You are not my father! Do not order me!* Then, the video freezes and a question pops up – *What would be Antonio’s best answer?* The participant will have three options – (1) *Punish Maria*; (2) *Ask Maria’s mother for help*; (3) *Explain to Maria what is his role within the stepfamily* and (4) *Watch the video again*. If the participant chooses option one (wrong option), he/she will see a video with António punishing Maria and Maria denying him authority. Then, the video freezes and a psychoeducational massage will appear – *initially, discipline and authority must be imposed by the biological parent; Maria and António have not yet developed a trust relationship, because of that, she has difficulties in recognizing his authority*. Option two is the “not the best option.” If selected, another psychoeducational massage arises – *While it is very important for the biological parents to support the decision of the stepparents, it is even more important for the stepparents to clearly assume their role within the family. How could António assertively tell Maria what is his role in the family?* Only option three is correct and if participant choose it, he/she will see the correct video with the correct behavior and the Game goes forward (see the sequence of images in Figure 4). Participants are encouraged to finish all modules and have the opportunity to communicate with a mental health professional for clarification of doubts or emotional support, if desired. The Game platform is checked daily to monitor participation. Participation reminders are sent weekly to finish the *GSteps* in the allotted time – 1 month.

*Wait-list*. After replying to the baseline questionnaire (*T_0_*), participants assigned to this condition receive a message informing that they are on the waiting list to participate on the course. The wait-list CG is also notified that they will be receiving further instructions in 1 month, and that access to the course is be possible after replying to a second assessment (*T_1_*).

## Data and statistical proposed analysis

Data will be analyzed using the Statistical Package for Social Sciences (SPSS), v.24 (IBM Corp, 2016). Preliminary and descriptive statistics will be conducted to describe the sociodemographic characteristics of the participants, standard deviations (SDs) and data normality. Missing data will be manage through intention-to-treat analysis (ITT). The Chi-square (*χ*^2^) and the independent sample *t*-test will be used to detect significant differences between the EG and CG on the *T_0_* sociodemographic characteristics and psychosocial variables. To explore the effect of the intervention on psychosocial variables and intervention perceived benefits, researchers will conduct repeated measures mixed ANOVAs to analyze the interaction between groups (EG and CG) and time (baseline, post-test and follow-up for EG; baseline and post-test for CG). To analyze the main effects, *post hoc* tests using Bonferroni correction will be performed. The independent *t* test will be used to compare how satisfied the EG and the CG are with the psychoeducational simulation Game on the post-intervention assessment (*T_1_*).

### Power analysis

G*Power (Faul et al., 2007) was used to calculate a minimum sample size. To test the efficacy of the intervention compared with the control condition, we propose a power analysis based on a probability level of 0.05 and a power of 0.80 (Hawkins et al., 2008; Lucier-Greer and Adler-Baeder, 2012). For analysis of covariance (ANCOVA), G*Power indicated an entire sample of 74 to detect a medium effect size of *d* = 0.5. However, a high dropout rate (about 50%; Wangberg et al., 2008) is usually encountered in internet-based interventions and thus we ultimately plan for a sample of 112 participants (56 participants per condition).

## Discussion

This protocol presents a RCT aimed at evaluating the efficacy of a web-based psychoeducational intervention (*GSteps*) designed for improving marital, (step)parenting and co-parenting skills in adults who live in stepfamilies. The use of this protocol could lead to the first web-based RCT study on (step)parenting and marital outcomes for Portuguese remarried people. The intervention and protocol could also be translated into other languages. *GSteps* includes content on stepparenting issues along with psychoeducational information related to the unique aspects of the remarriage spousal subsystem. This includes factors like fantasies and myths regarding remarriage, “emotional divorce” difficulties, losses normalization by the first-time marrying partner, financial management in the context of additional financial obligations such as alimony.

Intervention programs that include not only parental issues but also marital issues have shown greater improvements in individual, couple, family, and parenting functioning (Lucier-Greer et al., 2014). Regarding online interventions to remarried people, a study (Gelatt et al., 2010) that test the efficacy of a family life education program for stepfamilies that is self-administered, interactive, and web-based also found significant improvements in parenting and family domain. In fact, a review study of online learning studies revealed that learning outcomes for adults who engaged in online learning exceeded those of adults who received face-to-face instruction (U.S. Department of Education, 2009). Our psychoeducational intervention has the particularity of resorting to real-life simulation through an interactive Game. Simulation-based learning provide learning spaces in which learners can safely and repetitively practice and can be more effective than traditional approaches (Wayne et al., 2005; Bruce et al., 2009; Szögedi et al., 2010).

Then, if the *GSteps* has positive outcomes on marital adjustment, marital social skills, stepparenting and co-parenting attitudes, remarriage beliefs, stepfamily functioning and knowledge learned, this research will contribute to evidence on the efficacy of using internet platform to support stepfamilies. Besides that, this psychoeducational simulation Game could become a health care tool for health professionals to enhance stepfamily functioning, (step)parenting ability and marital adjustment of remarried adults.

## Strengths and limitations

Several strengths of the protocol and its design must be highlighted. This is a program that focuses on adults in stepfamilies, a vulnerable and understudied group, namely in the Portuguese context. This protocol underlines the importance of adapting psychoeducational intervention programs to the current demands of everyday life, namely, the exponential use of technological and virtual resources. Furthermore, literature suggest that online interactive multimedia programs can offer effective delivery of general education content (Cairncross and Mannion, 2001; Gelatt et al., 2010). As with other web-based interventions, this approach has a brief format, is low-cost and has a broad reach. The existence of subtitles in all videos enhances this reach and makes it possible to adapt the *GSteps* to other languages. Furthermore, it is a very comfortable type of intervention in which participants can receive the intervention from computer-devices in their own homes.

Moreover, by performing repeated measurements of psychosocial variables related with marital domain (dyadic marital adjustment; marital social skills; remarriage beliefs), parenting domain (SAB and CAB) and family functioning through a longitudinal and RCT design, this research protocol facilitates more reliable data on the outcome effects of the intervention. There are also some limitations of the protocol. All included measurement instruments are self-reports that can lead to a response-set tendency. Nonetheless, the majority of instruments are standardized inventories with good levels of reliability and validity or instruments that are tailor-made for the protocol. An expected limitation is the dropout rate during the intervention process as well as a significant missing data to follow-up assessment. To minimize this limitation, participants should be regularly notified by email to continue/end the *GSteps* or to participate in the post-and follow-up assessments.

### Dissemination

The use of this protocol could lead to publishable results in peer-reviewed scientific journals. Results could also be disseminated at national and international conferences or seminars. The more *GSteps* is known in the context of local institutions (social security, health centers) the more it can be accessed by stepfamilies.

## Conclusion

This protocol describes the development of a web-based psychoeducational intervention program (*GSteps*) which aims to improve marital, (step)parenting and co-parenting skills in adults who live in stepfamilies (parents and stepparents). The protocol also outlines a RCT study design to evaluate whether *GSteps* is an effective psychoeducational tool. The results of a RCT study could provide evidence of the efficacy brief, virtual training tools for stepfamilies. If proven efficacious, the implementation of *GSteps* could be explored in the clinical, social and health context.

## Ethics statement

The development of the program and protocol was approved by the Ethics Committee of the Faculty of Psychology and Education Sciences University of Porto (2019/4-2), conducted according to the Declaration of Helsinki for Medical Research Involving Human Subjects and registered in Clinical Trials.Gov (NCT05281913). All participants will be volunteers and data collection will begin after detailed information and signing a virtual informed consent. Participants will know that they have the possibility of ending their participation at any time without any implications. This study will comply the General Data Protection Regulation (GDPR) of the European Union. In the presentation of project results, no individuals will be identified ensuring data confidentiality. Possible risks, such as emotional or psychological discomfort, will be suppressed with the support of a mental health professional.

## Author contributions

CS is the principal investigator of this study and was primarily responsible for the design and development of the RCT. MM, contributed to the study design, drafting, and editing of the manuscript, BH and MC contributed to editing the manuscript and approved the final version.

## Funding

The author(s) declare that financial support was received for the research and/or publication of this article. This work was supported by the Portuguese Science and Technology Foundation under the project PD/BD/143069/2018, and by the Centre for Psychology at the University of Porto (CPUP), under the strategic funding UIDB/00050/2020.

## Conflict of interest

The authors declare that the *GSteps* intervention and protocol were designed in the absence of any commercial or financial relationships that could be construed as a potential conflict of interest.

## Correction note

A correction has been made to this article. Details can be found at: 10.3389/fpsyg.2025.1692245.

## Publisher’s note

All claims expressed in this article are solely those of the authors and do not necessarily represent those of their affiliated organizations, or those of the publisher, the editors and the reviewers. Any product that may be evaluated in this article, or claim that may be made by its manufacturer, is not guaranteed or endorsed by the publisher.
