# Correction: A web-based psychoeducational simulation game for adults in stepfamilies (*GSteps*)—study protocol for a randomized controlled feasibility trial

**DOI:** 10.3389/fpsyg.2025.1692245

**Published:** 2025-09-16

**Authors:** Carina Mota Santos, Maria Emília Costa, Brian Jensen Higginbotham, Mariana Veloso Martins

**Affiliations:** ^1^Faculty of Psychology and Education Science, University of Porto, Porto, Portugal; ^2^Center for Psychology, Faculty of Psychology and Education Science, University of Porto, Porto, Portugal; ^3^Utah State University, Logan, UT, United States; ^4^Emma Eccles Jones College of Education and Human Services, Utah State University, Logan, KS, United States

**Keywords:** remarriage, stepfamilies, web-based intervention, psychoeducational game, behavior modeling, randomized controlled trial, study protocol

In the published article, there was an error in the Funding statement. This work was supported by Portuguese Science and Technology Foundation under the project PD/BD/143069/2018.

The correct Funding statement appears below.

